# Supplementary material for: Seminoma and Embryonal Carcinoma Footprints Identified by Analysis of Integrated Genome-Wide Epigenetic and Expression Profiles of Germ Cell Cancer Cell Lines
Source: PLoS One. 2014 Jun 2;9(6):e98330. doi: 10.1371/journal.pone.0098330 (PMC4041891; doi:10.1371/journal.pone.0098330)
Supplement: File S1 — ZIP file containing DMRforPairs output for significant regions. Please start from the html files. (ZIP) [file pone.0098330.s008.zip › figures/29572.pdf]

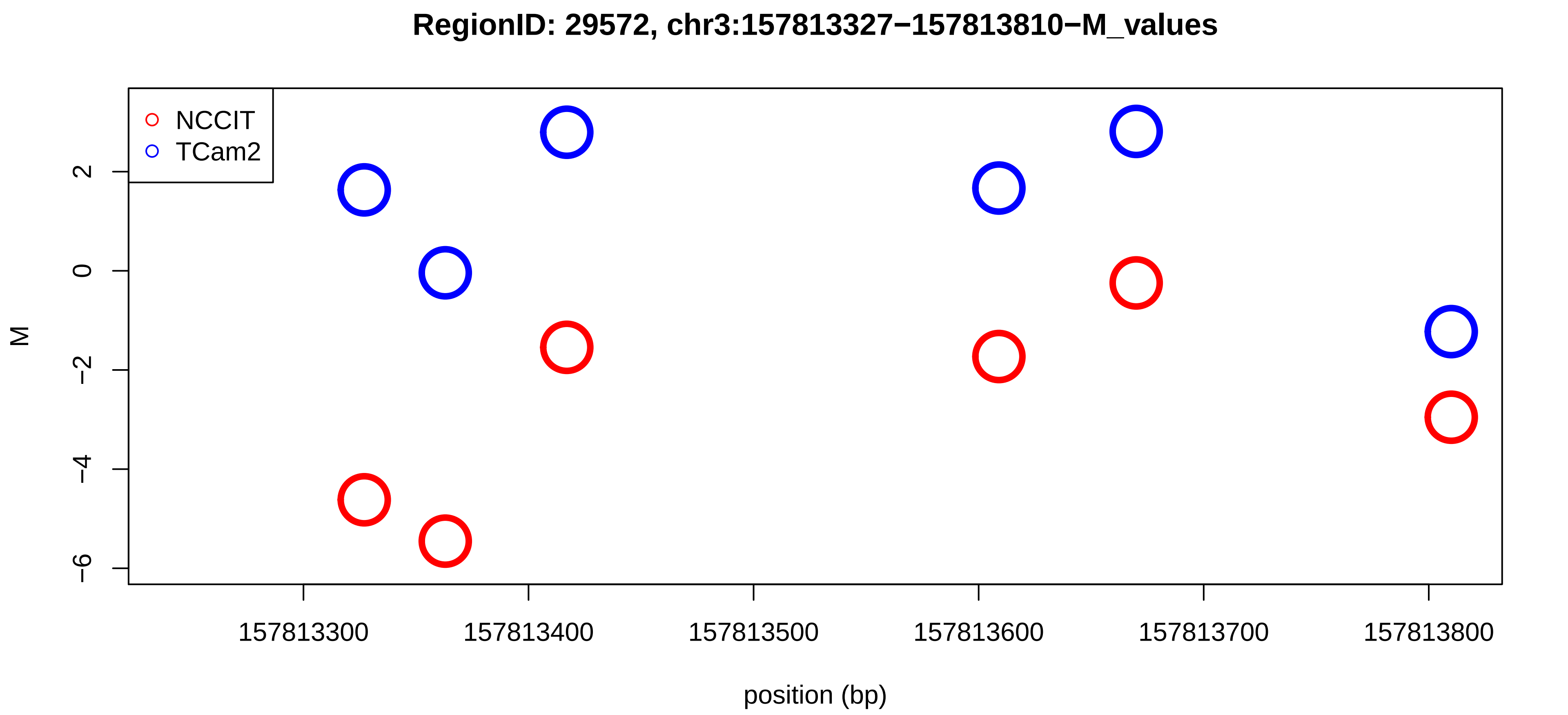
RegionID: 29572, chr3:157813327−157813810−M_values
NCCIT
TCam2
M
2
0
−2
−4
−6
157813300
157813400
157813500
157813600
157813700
157813800
position (bp)

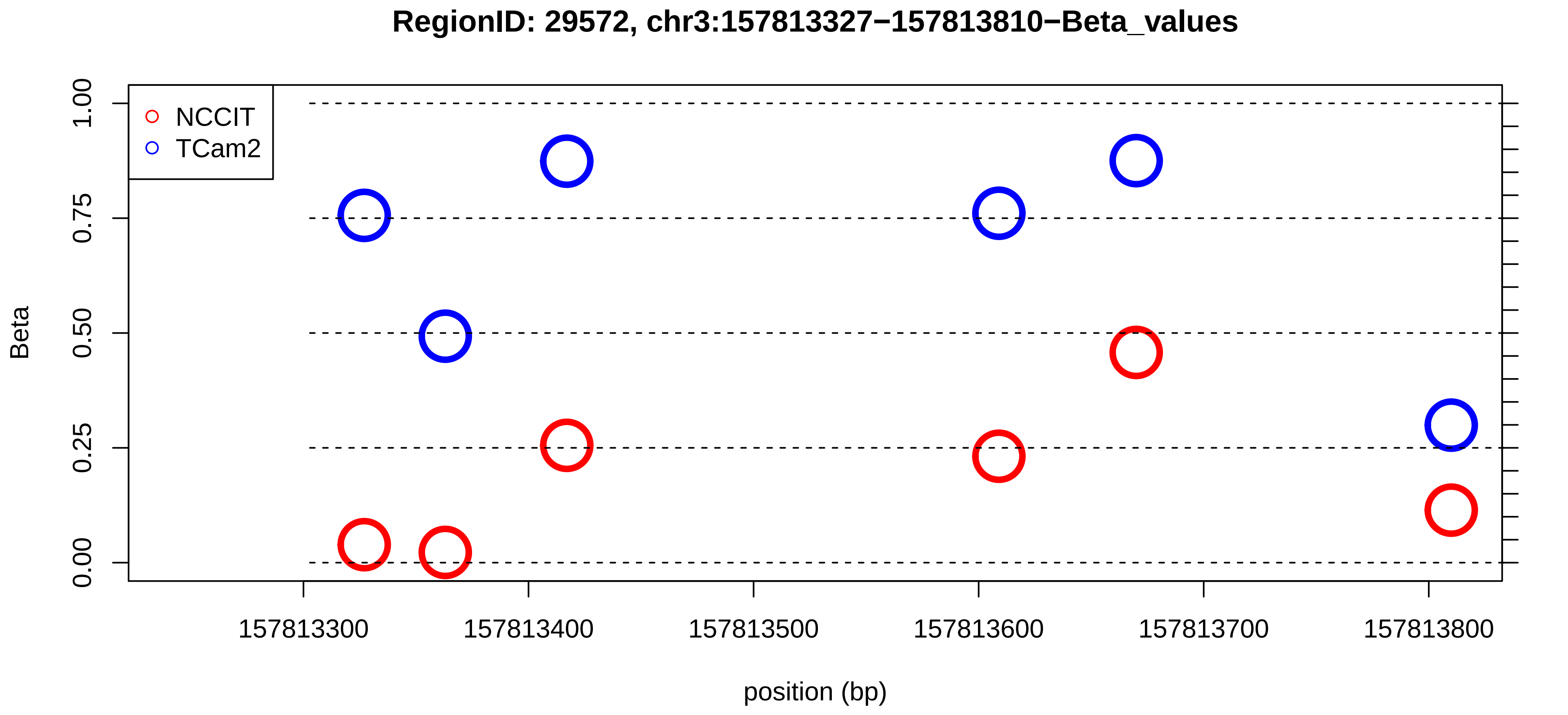
RegionID: 29572, chr3:157813327−157813810−Beta_values
NCCIT
TCam2
Beta
1.00
0.75
0.50
0.25
0.00
157813300
157813400
157813500
157813600
157813700
157813800
position (bp)
